# Hidden Dangers: A Gastrointestinal Stromal Tumor Concealed Inside of a Meckel's Diverticulum

**DOI:** 10.7759/cureus.24810

**Published:** 2022-05-07

**Authors:** Ami K Patel, Matthew Boykow, Neil Sondhi, James Garizio, Antonios Tsompanidis, Devarajan P Iyengar

**Affiliations:** 1 Internal Medicine, Rowan School of Osteopathic Medicine, Stratford, USA; 2 Internal Medicine, Bayonne Medical Center, Bayonne, USA; 3 Family Medicine, Bayonne Medical Center, Bayonne, USA; 4 Oncology, Bayonne Medical Center, Bayonne, USA

**Keywords:** bacterial peritonitis, small-bowel obstruction, meckel´s diverticulum, gastrointestinal stromal tumor (gist), perforated meckel's diverticulum

## Abstract

Meckel's diverticulum (MD) is one of the most common congenital abnormalities of the gastrointestinal tract, affecting approximately two percent of the population. Rarely, Meckel’s diverticula have been found to harbor various tumors, which go unnoticed until later in their course. The clinical presentation varies among each individual, and tumors have often metastasized or caused diverticular rupture at the time of diagnosis. This is a case of a 55-year-old male with a past medical history of alcohol abuse and asthma who presented to the emergency department with abdominal pain. He denied any fever, chills, chest pain, nausea, changes in urinary patterns, recent travel, or sick contacts. He is a non-smoker but has been a heavy drinker for many years. On physical exam, he was found to have diffuse abdominal tenderness with pain greatest in the epigastric region and no bowel sounds. He was afebrile but tachycardic at 112 bpm, hypertensive at 168/98 mmHg, and tachypneic at 38 bpm. Labs showed a markedly elevated white blood cell count, hemoglobin and platelet count, as well as metabolic acidosis and elevated lactate levels. Abdominal CT showed a mechanical small bowel obstruction with unclear etiology. Of note was a 7.2 cm thick-walled collection in the right lower quadrant having no clear communication with any bowel loops. Despite aggressive hydration and supportive care, his abdominal exam continued to worsen, prompting an exploratory laparotomy. During the laparotomy, a perforated MD with frank succus was found. On pathology, the affected segment of the bowel revealed a CD117 and CD34 positive spindle cell gastrointestinal stromal tumor (GIST) with mild cytological atypia, no necrosis, and no regional lymph node involvement. Cultures of peritoneal fluid were positive for *Klebsiella oxytoca*, and the patient was started on meropenem and doxycycline. The patient showed significant improvement with the appropriate administration of antibiotics and was eventually discharged to follow-up with hematology/oncology as an outpatient for further management and monitoring of his GIST tumor. This case is unique as there are only a few reported cases of patients developing GIST inside of MDs. Despite the high five-year survival rate of typically localized GIST tumors, the complications (such as perforation in the case of our patient) caused by tumor growth inside a MD are detrimental if not diagnosed promptly. Not only does perforation increase the risk of metastasis but also the risk of peritonitis and other complications. This case calls for more research on standardization of care for patients who have MD to prevent malignant transformations as well as potential prophylactic excision of incidental MD findings in adult patients.

## Introduction

Meckel's diverticulum (MD) is one of the most common congenital abnormalities of the gastrointestinal tract, affecting approximately two percent of the population [1,2]. These diverticula are formed because of inadequate closure of the vitelline duct at the fifth week of fetal growth and are true diverticula as they encompass all three layers of the mucosa [3]. This results in an outpouching of the small intestine, containing tissues normally found in the stomach and pancreas. Despite being among the most prevalent congenital abnormalities, it is often difficult to diagnose since many patients remain asymptomatic [1]. These diverticula are often found incidentally, especially in adults. This abnormality has also been associated with many life-threatening complications such as hemorrhage, intussusception, intestinal obstruction, and perforation [4,5]. These complications are more prevalent during the early years of life, which makes prophylactic excision of MD beneficial for younger patients [6]. Since the rate of complications has been found to be inversely proportional to age, prophylactic excision in older adults remains a heavily debated topic [6]. However, neoplasm formation within MD appears to occur more frequently as patients age [7].

Meckel’s diverticula have generally been considered benign, although a small percentage (0.5%-3%) of these have been found to harbor tumors of various origins [8]. In those who develop tumors, carcinoid tumors are the most prevalent, affecting 33%-44% of patients [8]. Slightly less prevalent are leiomyosarcoma (18%-25%), adenocarcinoma (12%-16%), and lastly, gastrointestinal stromal tumors (GISTs), which represent only 12% of tumors [8].

We present a case of a patient who underwent an exploratory laparotomy for an acute abdomen. Laparotomy revealed a perforated MD caused by spindle cell GIST, secondarily leading to bacterial peritonitis.

## Case presentation

**Figure 1:**
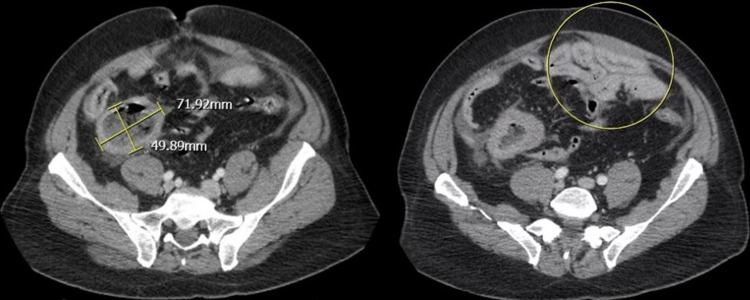
A CT scan of the abdomen shows that there is a bowel obstruction and a 7.2 cm collection in the right lower quadrant of the abdomen. There is no clear communication between the bowel loops. The arrow on the left shows the 7.2 cm collection and the circle on the right shows the bowel segments w/o communication with the rest.

This is a case of a 55-year-old male with a past medical history of alcohol abuse and asthma who presented to the emergency department with abdominal pain. He ate a bowl of chicken soup at midnight, and five hours later, he woke up with severe, squeezing abdominal pain, rated at 10/10. The pain was constant and did not radiate. The patient's last bowel movement was 12 hours before the presentation, and he reports no stool abnormalities. He also denied any previous episodes like this in the past. The patient denied fever, chills, chest pain, recent travel, sick contacts, nausea, or changes in urinary habits. He had no prior surgeries. He is a non-smoker and denies drug use, but he has been using alcohol heavily for 20 years. On admission, lipase was within normal limits at 56 U/L. On physical exam, he was found to have diffuse abdominal tenderness with distension, with pain greatest in the epigastric region. Bowel sounds were noted to be absent. The physical exam was otherwise unremarkable. He was afebrile, but was tachycardic at 112 bpm, hypertensive at 168/98 mmHg, and tachypneic at 38 bpm. Labs on admission showed elevated hemoglobin and platelet counts, normal white blood cell count (WBC), along with metabolic acidosis and elevated lactate levels (Table 1). Blood cultures taken at admission showed no bacterial growth. Abdominal CT showed a mechanical small bowel obstruction with unclear etiology. Of note, there was a 7.2 cm thick-walled collection in the right lower quadrant of the abdomen with no clear communication with bowel loops (Figure 1). The findings were suggestive of a perforated abscess. Despite aggressive hydration, nasogastric tube placement, and IV vancomycin, his abdominal exam worsened over the next 10 hours, prompting explorative laparotomy. Laparotomy revealed a perforated Meckel’s diverticulum in the right lower quadrant with frank succus. Purulent peritoneal fluid was collected and sent for culture. The 15 cm long affected segment of the bowel was resected and sent to pathology. Pathologic evaluation elucidated a grade one CD117 and CD34 positive spindle cell GIST with mild cytological atypia, no necrosis, and no regional lymph node involvement. The tumor nodes and metastases (TNM) classification of the tumor was pT3, pN0, and pM0. The increasing WBC prompted repeat blood cultures along with urine and nares, all of which were negative for pathology. Cultures of the peritoneal fluid were positive for *Klebsiella oxytoca*, and a chest x-ray was suspicious for hospital-acquired pneumonia. The patient was subsequently treated with varying durations of meropenem 1 gm Q8H IV, doxycycline 100 mg Q12 orally, ceftriaxone 2 mg daily IV, metronidazole 500 mg Q8H orally, and piperacillin/tazobactam 4.5 gm Q8H IV. He showed improvement after source control and continued antibiotic treatment, and hematology/oncology was consulted for the diagnosis of GIST. He was eventually stable for discharge on day 10 and was instructed to follow-up as an outpatient for further management of his GIST tumor. 

**Table 1:** Laboratory testing results at the time of admission, on days 2, 4, and 8 of hospitalization, and at discharge on day 10. WBC: white blood cells, Hgb: hemoglobin, BUN: blood urea nitrogen, AST: aspartate aminotransferase, and ALT: alanine transaminase.

Measures	Admission	Day 2	Day 4	Day 8	Discharge
WBC (10^3^ u/L)	9.0	12	16.0	22.4	15.1
Hgb (g/dL)	18.6	14.6	11.2	12.9	11.2
Platelets (10^3^ u/L)	582	390	382	577	562
Lactate (mmol/L)	5.0	2.9	0.9	-	-
BUN (mg/dL)	21	14	16	17	13
Total bilirubin (mg/dL)	1.6	0.8	0.9	0.8	0.7
AST (U/L)	34	33	36	43	32
ALT (U/L)	21	16	13	13	11
Alkaline phosphatase (U/L)	56	44	106	164	141

## Discussion

This presentation is unique in that very few cases have been reported of patients developing GIST tumors in MD and those reported have all been clinically distinct. In fact, during our literature review, we only found 18 previous cases of GIST-associated perforation of MD. Typical GIST tumors are most often found to originate from either the stomach or the small bowel [9]. However, GIST originating from outside the stomach has been found to have a greater risk of progressing to malignancy, making early diagnosis crucial for these patients [10,11]. Little is known about the behavior of GIST tumors as most occur sporadically. However, in the majority of GIST tumors within MD, patients remain asymptomatic until the tumor grows larger than 5 cm [8]. Once they become symptomatic, patients commonly present with abdominal pain, nausea, vomiting, and weight loss [8]. Tumor diameter greater than 5 cm and a high mitotic count are also considered poor prognostic indicators [8]. Patients’ status post-operative GIST removal without adjuvant therapy has been found to have a greater risk of recurrence if they possess any of the following characteristics: male sex, large GIST tumors, high mitosis count, non-gastric primary location, or the presence of rupture [12]. The behavior of the GIST tumor in our patient is unable to be accessed as he did not have evidence of metastases and surgical intervention is thought to be curative at present.

Perforation of MD as observed in our patient is unusual, and if seen, it is likely secondary to a foreign body or diverticulitis and necrosis [13]. A potential mechanism in our patient could be GIST invasion into the muscularis propria, causing bowel necrosis and wall fragility [14]. Another mechanism could be increased pressure from the tumor, causing obstruction and leading to perforation. In our case, the latter is more likely given the large size of the tumor and the lack of necrosis observed. Of the cases reviewed, there was only one other case that mentioned the presence of bowel obstruction in the setting of MD perforation due to GIST. When presented with a patient with non-specific abdominal symptoms, it is important to keep GIST on the list of differential diagnoses as it can be easily misdiagnosed. 

## Conclusions

Current literature supports the removal of symptomatic MD in children due to higher complication rates, although there is controversy about prophylactic removal in adulthood. Generally, the rate of complications is lower in adults, making the benefits of intervention less clear. However, it is important to understand that the risk of neoplasm formation within MD occurs more frequently in patients as they age over 50. Thus, managing incidental findings of MD solely by observation can result in missing neoplasm formation. This is because many patients do not present with symptoms until later in their course when the tumor has increased in size, which ultimately increases the risk of complications such as perforation. Thus, if a patient presents with an abdominal mass, acute abdomen, and peritonitis, GIST preformation should be considered despite its low incidence due to its high morbidity. Further investigation is needed to determine the appropriate management of patients who are found to have MD in adulthood. This could lead to the prevention of forming such tumors and their complications. Therefore, better algorithms for screening or prophylactic diverticulectomy are needed to reduce the incidence of hidden tumors in this population. 
